# Tetraspanin CD53 Promotes Inflammation but Restrains Mucus Production in a Mouse Model of Allergic Airway Inflammation

**DOI:** 10.1111/all.16426

**Published:** 2024-12-09

**Authors:** Amy T. Hsu, Viktor Bugajev, Timothy A. Gottschalk, Livia Demkova, Lucie Potuckova, Lubica Draberova, Monika Bambouskova, Philipp Hagemann, Caitlin A. O'Brien, Martin Riecan, Ondrej Kuda, Evelyn Tsantikos, Petr Draber, Mark D. Wright, Annemiek B. van Spriel, Margaret L. Hibbs, Ivana Halova

**Affiliations:** ^1^ Department of Immunology Alfred Research Alliance, Monash University Melbourne Victoria Australia; ^2^ Laboratory of Signal Transduction Institute of Molecular Genetics of the Czech Academy of Sciences Prague Czech Republic; ^3^ Department of Medical BioSciences Radboud University Medical Center Nijmegen The Netherlands; ^4^ Laboratory of Metabolism of Bioactive Lipids Institute of Physiology of the Czech Academy of Sciences Prague Czech Republic

To the Editor,

CD53 is a pan‐leukocyte‐expressed tetraspanin that is implicated in controlling immune cell function, including leukocyte trafficking [1]. CD53 is associated with several inflammatory diseases such as asthma, where genetic studies report that a promotor polymorphism that reduces CD53 expression is associated with higher asthma risk [2]. Herein, the role of CD53 in mast cell responses and Type 2 inflammation was investigated.

Bone marrow‐derived mast cells (BMMC) from wild‐type (WT) and *Cd53* ^−/−^ mice activated with IgE antigen did not differ in early activation events (Figure S1A–H). Analysis of the late phases of IgE‐antigen activation revealed that neither prostaglandins nor leukotrienes were increased in the absence of CD53 (Figure 1A). However, proinflammatory cytokine production (Figure S1I) and secretion (Figure 1B) were significantly increased by CD53 deficiency. Expression of CD53 in *Cd53* ^−/−^ and WT BMMCs rescued TNF‐α overproduction (Figure 1C) directly implicating CD53 in this phenotype. Elevated cytokine production was FcεRI‐dependent, as no differences in cytokine production were observed in cells activated with thapsigargin (Figure S1J). Further analyses showed that CD53 deficiency augmented nuclear translocation of NF‐κB (Figure 1D,E) and prolonged phosphorylation of c‐Jun (Figure 1F).

**FIGURE 1:**
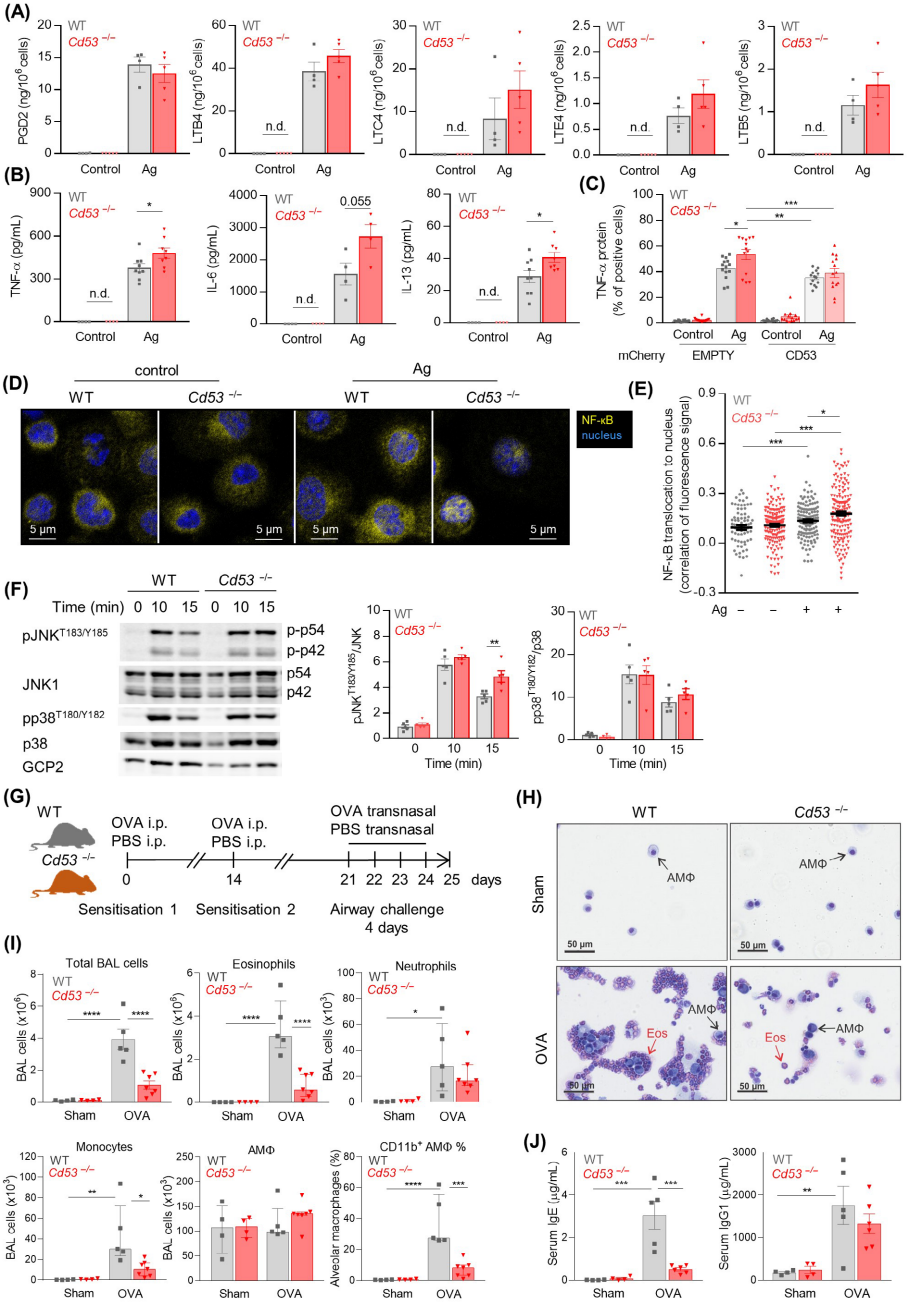
CD53 deficiency enhances FcεRI‐mediated cytokine production in BMMCs but suppresses cellular inflammation and IgE production in OVA‐challenged mice. (A–E) BMMCs were left unstimulated (Control) or activated with IgE‐Ag (Ag). (A) Prostaglandins and leukotrienes production. (B) Cytokine production. (C) TNF‐α production in WT and *Cd53* ^−/−^ BMMCs transfected with empty mCherry expression vector or mCherry expression vector with hCD53. (D, E) NF‐κB p65 subunit localization to the nucleus (15–50 cells per cell type and condition, *n* = 3); (F) Representative image and evaluation of JNK and p38 phosphorylation. GCP2 is independent loading‐control. (G–J) Evaluation of inflammation in sham or OVA‐challenged WT and *Cd53* ^−/−^ mice (*n* = 4–7). (G) Experimental design. (H) Cytospins of cells from bronchoalveolar lavage (BAL), and immune cell counts in BAL from flow cytometry and cell counts (I). (J) Serum IgE and IgG1 levels. *p < 0.05, **p < 0.01, ***p < 0.001, ****p < 0.0001.

Given these findings, we assessed the role of CD53 in allergic airway disease by immunizing and challenging mice with PBS (sham) or ovalbumin (OVA) (Figure 1G). In contrast to the exaggerated signaling and cytokine production observed in *Cd53* ^*−/−*^ BMMCs, components of the immune response were dampened in OVA‐challenged *Cd53* ^*−/−*^ mice, including a significant reduction in leukocyte recruitment to the lungs and airspaces, particularly eosinophils (Figure 1H,I, Figure S2A,B). While steady‐state IgE levels were not affected by CD53‐deficiency, IgE responses were increased > 200‐fold in OVA‐challenged WT mice but this response was markedly dampened in OVA‐challenged *Cd53* ^−/−^ mice, although conversely IgG1 levels were unchanged, suggesting impaired sequential class‐switching from IgG1 to IgE (Figure 1J, Figure S2D).

Airway hyperresponsiveness to inhaled methacholine revealed that respiratory system elastance and tissue elastance were increased in OVA‐challenged *Cd53* ^−/−^ mice at baseline and to low doses of methacholine, while baseline tissue damping was increased (Figure 2A–D). Airway mucus staining showed no significant differences between genotypes, but revealed a trend towards increased goblet cell numbers in OVA‐challenged *Cd53* ^−/−^ mice (Figure 2E,F). Expression of mucin genes and regulators (*Muc5ac*, *Muc5b, Clca1*) were markedly upregulated in OVA‐challenged *Cd53* ^*−/−*^ mice (Figure 2G,H), correlating with mildly enhanced mucus deposition in the large airways and dysregulated lung function. The classical mucin‐inducing cytokines, IL‐4 and IL‐13 [3], were variably increased in OVA‐challenged mice, but not significantly different between WT and *Cd53* ^−/−^ mice (Figure 2I). Two other mucin‐inducing cytokines, IL‐6 and TNF‐α [3], were trending or significantly increased in the lung wash of OVA‐challenged *Cd53* ^−/−^ mice (Figure 2I), although not to the levels expected to drive a strong mucus response, but this combination may underlie the observed phenotype in *Cd53* ^−/−^ mice. Other cytokines and chemokines were elevated in OVA‐challenged mice, but with the exception of IL‐1β and IL‐12p40, which were significantly reduced in the absence of CD53, no major differences were observed between WT and *Cd53* ^*−/−*^ mice (Figure S2C).

**FIGURE 2:**
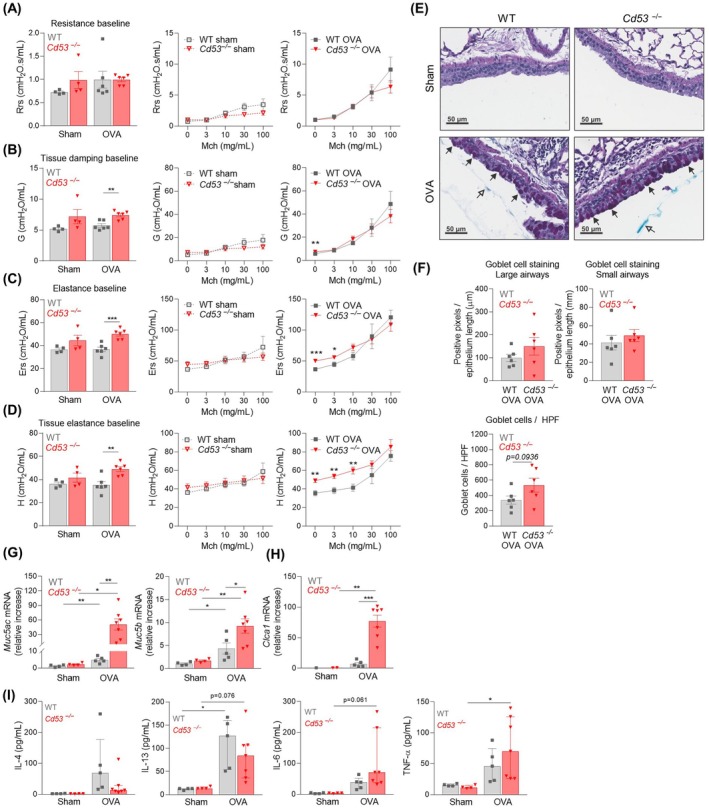
CD53 deficiency results in altered lung function at baseline and mucin production in allergen‐challenged mice. (A–D) Lung function parameters at baseline (no methacholine) in sham and OVA‐challenged mice (left graphs) and postmethacholine (Mch) challenge in sham (middle graphs) vs. OVA‐challenged mice (right graphs) assessing (A) respiratory system resistance [Rrs], (B) tissue damping [G], (C) respiratory system elastance [Ers], and (D) tissue elastance [H]. (E) Goblet cell metaplasia and mucus in the large airways. Closed arrows = goblet cells, open arrows = mucus. (F) Goblet cell quantification in large and small airways and per high‐power field (HPF). Expression of (G) mucin genes and (H) *Clca1* in lung tissue by qPCR. (I) Protein concentrations of mucin‐stimulating cytokines in BAL fluid measured by multiplex assay. *n* = 4–7, *p < 0.05, **p < 0.01, ***p < 0.001.

Although CD53 expression is thought to be restricted to hematopoietic cells [4], a recent report shows an important role for CD53 in metabolic signaling in hepatocytes [5]. This raises the possibility that CD53 may have a non‐immune cell function in the lung. According to the Human Protein Atlas, CD53 is expressed in type II alveolar epithelial cells and by ciliated and club cells in the airways. Thus, deficiency of CD53 may directly promote mucin gene expression in airway epithelium. Interestingly, there is a precedent for another tetraspanin, TSPAN8, regulating goblet cell metaplasia and mucus secretion [6]. Future studies should focus on assessing the role of CD53 in these key non‐immune cells.

Despite numerous studies, the role of mast cells in mouse models of allergic asthma remains controversial [7, 8]. Our results, showing that CD53‐deficiency led to reduced airway inflammation in vivo but increased proinflammatory cytokine production in mast cells in vitro, may seem contradictory. However, they suggest a multifunctional role for CD53 in different cell types and processes. Increased proinflammatory cytokine production in vitro aligns with previous finding in a macrophage cell line after CD53 knockdown [2]. Although reduced cellular inflammation was apparent in the lungs of OVA‐challenged *Cd53* ^−/−^ mice, allergen‐induced cytokines in BAL fluid, including those produced by mast cells, were not largely different. Diminished leukocyte migration into inflamed lungs corroborates previous reports of impaired *Cd53* ^−/−^ leukocyte recruitment [1, 9], while impaired IgE responses correlates with reduced humoral immunity in *Cd53* ^−/−^ mice [9]. No significant differences in mast cell numbers between sham and OVA‐challenged mice of either genotype was observed (Figure S2B), which may be due to the chosen immunization schedule. Mast cells are one of several sources of cytokines in vivo, and whether CD53 also regulates proinflammatory cytokine production in Th2 cells, eosinophils or basophils remains to be determined.

In conclusion, this study demonstrates that CD53 has a complex and multifaceted role in asthma, being required for inflammatory leukocyte recruitment to asthmatic lungs and the production of IgE, yet negatively regulating mast cell signaling, cytokine and mucus production.

## Author Contributions

A.T.H.: methodology, formal analysis, investigation, writing. V.B., T.A.G., Li.D., L.P., Lu.D., M.B., P.H., C.A.O., E.T., M.R. and O.K.: methodology, formal analysis, investigation. P.D., M.D.W., A.B.S. and M.L.H.: conceptualization, funding acquisition, supervision, writing. I.H.: conceptualization, methodology, formal analysis, investigation, funding acquisition, writing.

## Ethics Statement

All mouse studies complied with European legislation (directive 2010/63/EU of the European Commission) and were approved by local authorities (CCD, The Hague, the Netherlands), Institutional Animal Ethics Committee Review Board, IMG of the CAS guidelines (permit number 12135/2010–17210), and Czech national guidelines (2048/2004–1020). In vivo asthma studies were approved by the Alfred Research Alliance Animal Ethics Committee (E‐1752‐2017M, Australia). All experiments were conducted in accordance with the ARRIVE guidelines.

## Conflicts of Interest

The authors declare no conflicts of interest.

## Supporting information


Data S1

Data S2

## Data Availability

The data that support the findings of this study are available from the corresponding author upon reasonable request.
