# Supplementary material for: Tetraspanin CD53 Promotes Inflammation but Restrains Mucus Production in a Mouse Model of Allergic Airway Inflammation
Source: Allergy. 2024 Dec 9;80(4):1127–31. doi: 10.1111/all.16426 (PMC11969303; doi:10.1111/all.16426)
Supplement: Supplementary file 1 — Data S1 [file ALL-80-1127-s001.docx]

**Tetraspanin CD53 promotes inflammation but restrains mucus production in a mouse model of allergic airway inflammation**

**Materials and methods**

**Animals and cells:** CD53-deficient (*Cd53*^-/-^) mice have been described.^1^ These mice were bred on a C57BL/6J background, and age/sex matched wild-type controls were used in all experiments (*Cd53*^+/+^ or C57BL/6: WT). Mice were fed a standard diet with freely available water and food.

**Mast cell activation and cultivation:** Bone marrow cells were isolated from femurs and tibias of 8- to 14-week-old WT and *Cd53*^-/-^ littermates and cultured to obtain mature bone marrow-derived mast cells (BMMCs) as described.^2^ Analysis of **mast cell activation** was done as described.^2^ Briefly, BMMCs were sensitized for 16 h with 1 μg/mL 2,4,6-trinitrophenol (TNP)-specific mouse IgE (IGEL b4 1)^3^ in the absence of SCF and IL-3. Cells were washed with buffered saline solution (BSS; 20 mM HEPES, pH 7.4, 135 mM NaCl, 5 mM KCl, 1.8 mM CaCl_2_, 5.6 mM glucose, 1 mM MgCl_2_) supplemented with 0.1% fatty acid-free and globulin-free bovine serum albumin (BSA; Sigma, Cat. No. A7030) and activated with antigen (250 ng/ml, TNP-BSA conjugate, 15–25 mol TNP/mol BSA, prepared in our laboratory) or 1 µM thapsigargin (Invitrogen, Cat. No. T7459) at 37°C for indicated times: β-glucuronidase release - 30 minutes, Ca^2+^ release - continuously 0-5 minutes, protein phosphorylation – 0, 5, 10, 15 minutes, mRNA cytokine production – 1 hour. Concentrations of **secreted cytokines** were measured from cell-free supernatants of 5 hours activated and non-activated BMMCs using a bead-based immunoassay (LEGENDplex Mouse B Effector Panel; BioLegend, Cat No. 740820) according to the manufacturer's instructions. Aliquots of the same supernatants were used for the measurements of **secreted prostaglandins and leukotrienes** by mass spectrometry as previously described.^4^

**Cytokine production "rescue" experiments:** For rescue experiments, hCD53 fused to pmCherry^1^ or empty pmCherry-C1 was introduced into BMMCs using the Amaxa Nucleofector II (Lonza, Germany) according to the manufacturer′s instructions (program Y-001). Transfected cells were sensitized for 4 hours with IgE in the presence of brefeldin A (5 μg/ml) and activated with Ag in the presence of brefeldin A (5 μg/ml) for 2.5 hours. Cells were fixed with 4% paraformaldehyde in PBS for 15 min at 22°C. After washing in PBS, cells were permeabilized with 0.1% saponin in PBS for 15 min at 22°C. Cells were stained with anti-TNF-α BV421-conjugated Ab for 1 h and analyzed by flow cytometry. Cells were pre-gated for mCherry positivity and the percentage of TNF-α positive cells was calculated.

**Translocation of NF-κB** p60 subunit to the nucleus upon IgE-antigen activation was performed and analyzed as described.^5^ Briefly, IgE-sensitized cells were attached to fibronectin-coated slides and activated with Ag for 30 minutes. Fixed and permeabilized cells were stained with rabbit Abs against p65 subunit of NF-κB (1:400, #8242 and 1:200, #sc-372), followed by donkey anti-rabbit-Alexa Fluor 488 conjugate (1:200) and visualized by confocal microscopy.

**Passive systemic anaphylaxis.** Male mice aged 11 to 16 weeks were sensitized by intraperitoneal injection with TNP-specific IgE (45 µg in 400 µl of PBS) and 24 h later, anaphylaxis was induced by tail vein injection of antigen (TNP-BSA; 100 μg in 200 μl PBS/20 g mouse). Body temperature was recorded at 10-min intervals for at least 2.5 hours after antigen challenge.

**Ovalbumin (OVA)-induced mouse asthma model.** 8–9-week-old C57BL/6 (WT) and *Cd53^-/-^* mice were challenged using an OVA model of allergic asthma (n = 4-7 in two biological replicates; Figure 1F). Mice were sensitized to OVA through i.p. injection of 20 μg OVA (Sigma-Aldrich, Darmstadt, Germany) mixed with 100 μl of the adjuvant Alum (Alhydrogel 2% adjuvant, InvivoGen, San Diego, CA, USA) on days 0 and 14. The mice were then challenged on days 21-24 by transnasal administration of 10 μg OVA to stimulate an allergic asthma response as described previously.^6^ Control (sham) mice received PBS sensitizations and challenges. Mice were euthanized and analyzed 24 hours after the last transnasal dose. At the mouse asthma study endpoint, **bronchoalveolar lavage** (BAL) and **lung tissue** were collected and processed for **flow cytometry** as described.^7^ Briefly, lung tissue was chopped into small pieces and digested with 0.1 mg/ml Liberase TL (Roche, Basel, Switzerland) and 40 μg/ml DNase I, grade II (Roche) at 37^o^C followed by a 30 second blitz on the GentleMACS dissociator (Miltenyi Biotec, Bergisch Gladbach, Germany). BAL and processed lung tissue cells were stained with the following antibodies: B220-BV786, CD3e-FITC, CD8a-PerCP-Cy5.5, CD11b-BV786, CD45-BUV395, Siglec-F-BV421, Strepavidin-BV711 (all BD Biosciences), TCR γ/δ-PE, CD4-BV510, I-Ab MHC II-PerCP-Cy5.5, Ly6G-BV510 (all Biolegend), CD11c-AlexaFluor700, CD44-APC-eFluor780, CD62L-PE-Cy7, CD115-biotin, Ly6C-APC-eFluor780, (all Thermo Fisher Scientific) and analyzed with an LSRFortessa X-20 (BD Biosciences, Franklin Lakes, NJ, USA). The data was then processed using FlowJo software. The gating strategies used for flow cytometry of BAL cells and lung tissue digests shown in Supplementary file – Western blot images and gating strategies.

**Lung function measurements** and methacholine challenges were performed using the SCIREQ flexiVent.^8^ Mice were anaesthetised with 125 mg/kg ketamine/12.5 mg/kg xylazine for surgery to cannulate the trachea. After tracheostomy, mice were given a terminal dose of ketamine/xylazine and the cannula attached to the flexiVent. Airway hyperresponsiveness was tested by administering aerosolised methacholine (Mch) in increasing doses (0, 3, 10, 30, 100 mg/ml) with the flexiVent Aeroneb ultrasonic nebuliser (SCIREQ), during which lung function parameters were measured and obtained.

**Lung histological analyses.** Lung tissue from the mouse asthma study was inflation-fixed with 10% neutral buffered formalin (NBF) for at least 24-hours, processed by the Monash Histology Platform (VIC, Australia) and embedded in paraffin. Four-micrometer sections were cut. PAS/AB and toluidine blue staining was performed by the Monash Histology Platform. PAS/AB-stained goblet cells were analyzed by (1) positive pixel counts using a macro designed on Aperio ImageScope (Leica Biosystems, Nussloch, Germany); (2) nuclei and staining analysis using HALO AI analysis software (Indica Labs, Albuquerque, NM, USA); and, (3) manual cell counting on high power field (HPF) images taken on the Aperio ImageScope.

**Multiplex assay.** Selected proteins in mouse BAL fluid were measured using a Bio-Plex Pro Mouse Cytokine 23-Plex Immunoassay #M60009RDPD (Bio-Rad, Hercules, CA, USA), and the plate was run on a Bio-Plex 200 System (Bio-Rad).

**Measurement of Ig levels by enzyme-linked immunosorbent assay (ELISA).** Briefly, 96-well plates (Nunc, Thermo Fisher Scientific) were coated with IgE or IgG1 capture antibody (BD Pharmingen, BD Biosciences) overnight at 4°C, then washed with wash buffer (0.05% Tween-20 in PBS) and blocked with Assay Diluent (BD Biosciences) for 1 hour. The plates were then washed and incubated with IgE or IgG1 standards (BD Pharmingen) and diluted serum samples for 2 hours. This was followed by washing and incubation with detecting antibody (BD Pharmingen) for 1 hour, followed by another wash and incubation with streptavidin-HRP (BD Pharmingen) for 30 minutes. After washing, TMB substrate (BD Biosciences) was added to the plates for a colorimetric reaction and the reaction was stopped with 1.5 M H_2_SO_4_. Plates were read on a Multiskan FC Microplate Photometer (Thermo Fisher Scientific) at 450 nm, subtracting at 595 nm. Standard curves were generated and the sample concentrations calculated using the SkanIt software (Thermo Fisher Scientific).

**Sequences of primers** used for qPCR evaluation in this article can be provided upon request.

**Statistical analysis.** Statistical analyses were performed using GraphPad Prism version 9.0.1 (GraphPad Software). Data are presented as mean ± SEM of at least two independent experiments and 4-10 biological replicates. Comparisons between groups were analyzed by Student's t-test (two groups), one-way analysis of variance (ANOVA) followed by Tukey's post-test (more than two groups). * p < 0.05, ** p < 0.01, *** p < 0.001 and **** p < 0.0001

**SUPPLEMENTARY FIGURES**

**
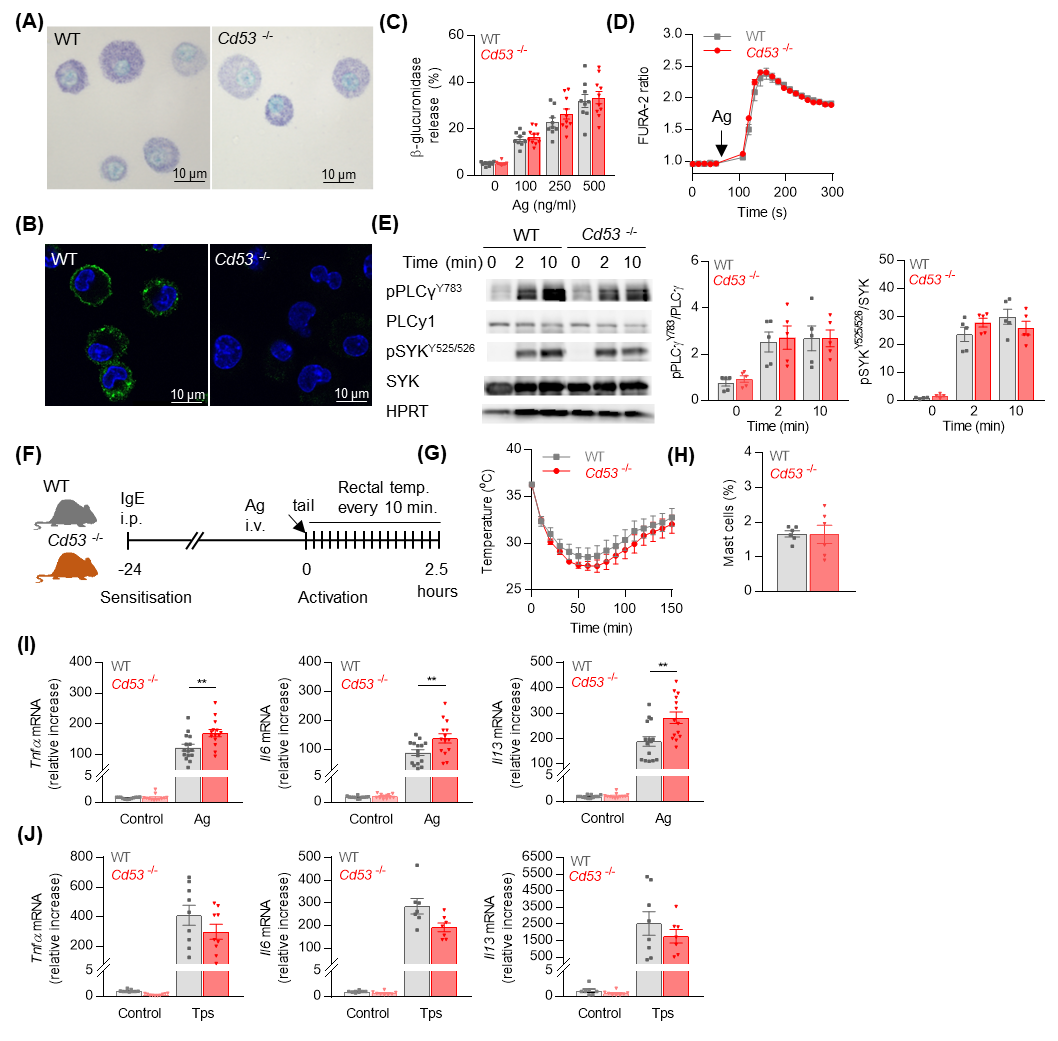
**

**Supplementary Figure 1.** *CD53 deficiency does not affect early activation events in mast cells but increases proinflammatory cytokine production upon IgE-Ag stimulation.* (A) Morphology of WT and *Cd53*^-/-^ BMMCs stained by toluidine blue. Magnification - 100x. (B) CD53 expression in mature BMMCs (blue-nucleus, green – anti-CD53-FITC). (C) BMMC degranulation as measured by the β-glucuronidase release. (D) Levels of free intracellular Ca^2+^ measured as changes in fluorescence in cells loaded with FURA-2 AM before and after Ag stimulation (arrow). (E) Immunoblot analysis with statistical evaluation of pSYK, pPLCγ and corresponding loading controls of BMMC lysates at different times after Ag stimulation. HPRT was used as an independent loading control. (F) Experimental design of passive systemic anaphylaxis (PSA). (G) PSA-induced changes in rectal temperature over a 2.5-hour period post-challenge. (n=9) (H) Percentage of mast cells in peritoneum. (I,J) Relative mRNA expression of *Tnf-α*, *Il-6* and *Il-13* in (I) non-activated and IgE-Ag activated or (J) non-activated and thapsigargin-activated (Tps) BMMCs. Results are represented as mean ± SEM. Each dot represents an individual biological replicate. Statistical significance was analyzed by Student *t*-tests at each indicated condition/time point, ** p < 0.01. Uncropped blots are available in supplementary file - Western blot images and gating strategies.

**
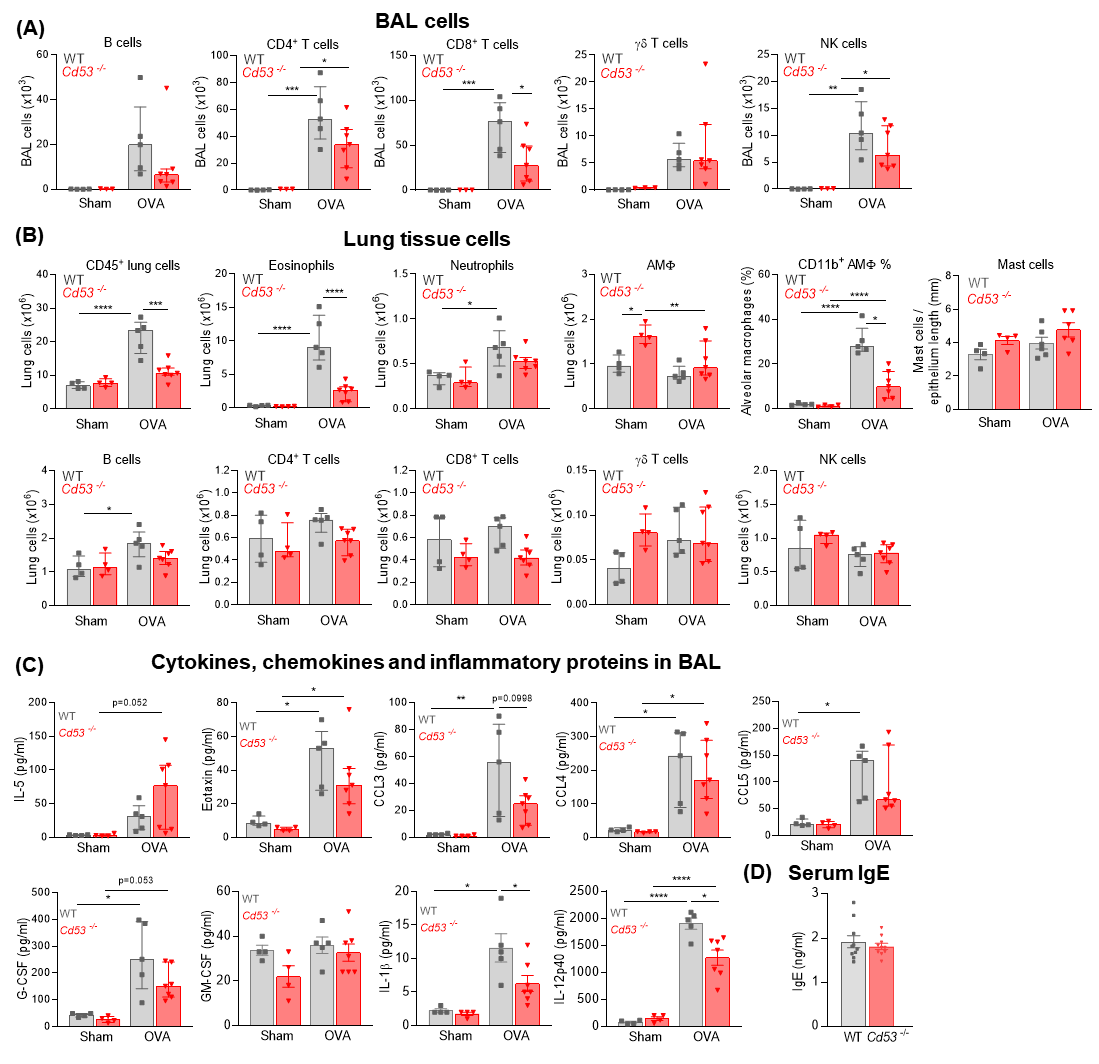
**

**Supplementary Figure 2.** *Absence of CD53 leads to reduced cellular inflammation in the lungs of allergen-challenged mice.* Cellular composition of (A) bronchoalveolar lavage (BAL) and (B) lung tissue of sham and OVA-challenged mice by flow cytometry and cell counts. Mast cells in Toluidine Blue-stained lung sections were manually counted. (C) Protein concentrations of cytokines, chemokines and inflammatory proteins of BAL fluid measured by multiplex assay. (D) Levels of total IgE in serum of non-immunized WT and *Cd53*^-/-^ mice. All results are presented as mean ± SEM. Each dot represents an individual biological replicate, n=4-7. Data was analyzed by ANOVA followed by Tukey's post-test * p < 0.05, ** p < 0.01, *** p < 0.001, **** p < 0.0001.

Gating strategies are available in supplementary file - Western blot images and gating strategies.

**Summary of the study**


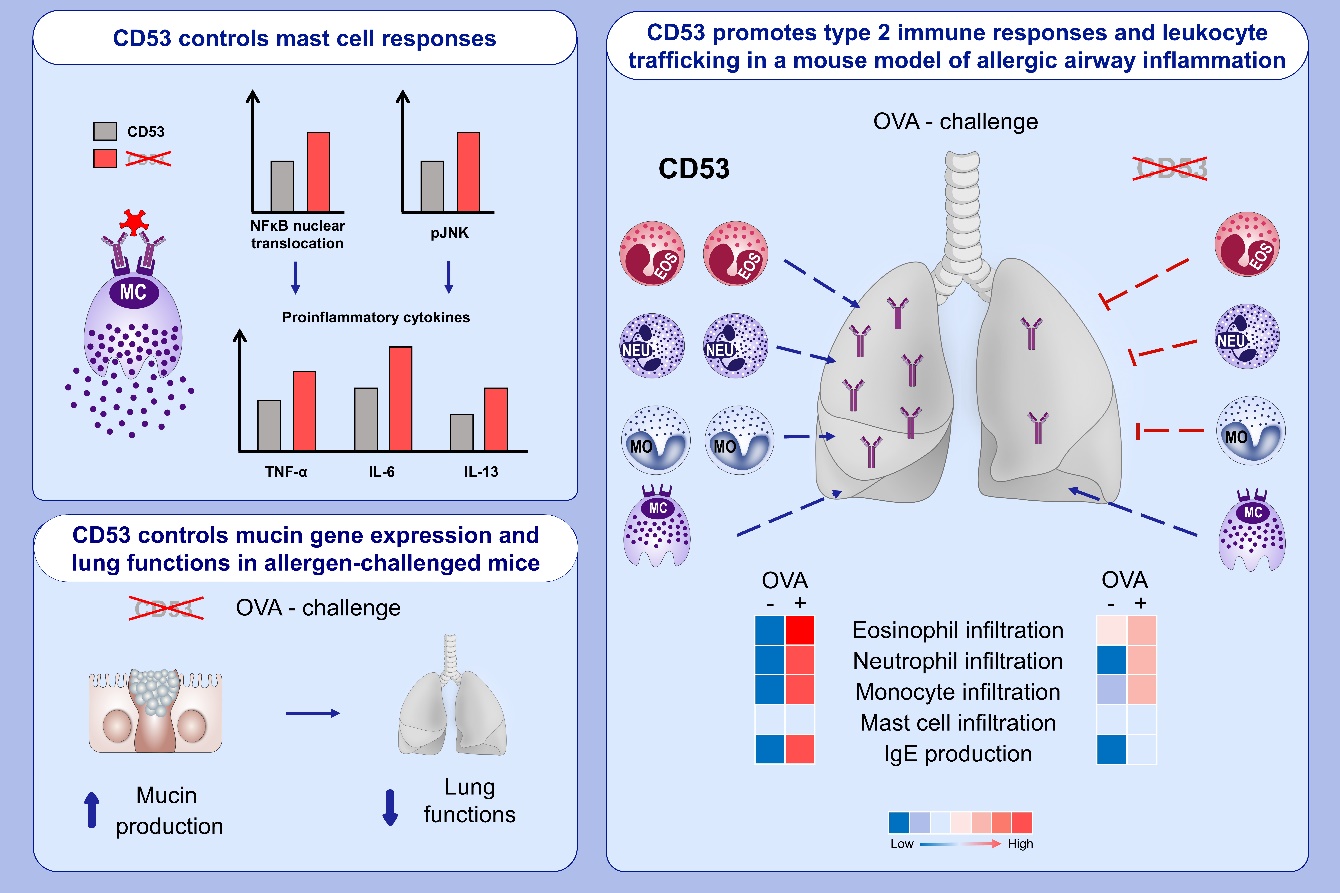


**HIGHLIGHTS**

- *Cd53*^-/-^ mast cells exhibit enhanced pro-inflammatory cytokine production due to exaggerated NF-κB activation and JNK phosphorylation.
- Tetraspanin CD53 promotes type 2 inflammation evidenced by dampened immune cell infiltration into the lungs of allergen-challenged *Cd53*^-/-^ mice and decreased production of IgE.
- Lung function is compromised in allergen-challenged *Cd53*^-/-^ mice, likely due to overproduction of mucins.

**Abbreviations:** EOS, eosinophil; IL, interleukin; IgE, immunoglobulin E; JNK, c-Jun N-terminal kinase; MC, mast cell; MO, monocyte; NEU, neutrophil; NF-κB, nuclear factor-kappaB; OVA, ovalbumin; TNF, tumor necrosis factor

**REFERENCES**

1. Zuidscherwoude M, Dunlock VE, van den Bogaart G, et al. Tetraspanin microdomains control localized protein kinase C signaling in B cells. *Sci Signal.* 2017;10(478).

2. Demkova L, Bugajev V, Utekal P, et al. Simultaneous reduction of all ORMDL proteins decreases the threshold of mast cell activation. *Sci Rep.* 2023;13(1):9615.

3. Rudolph AK, Burrows PD, Wabl MR. Thirteen hybridomas secreting hapten-specific immunoglobulin E from mice with Iga or Igb heavy chain haplotype. *Eur J Immunol.* 1981;11(6):527-529.

4. Bugajev V, Paulenda T, Utekal P, et al. Crosstalk between ORMDL3, serine palmitoyltransferase, and 5-lipoxygenase in the sphingolipid and eicosanoid metabolic pathways. *J Lipid Res.* 2021;62:100121.

5. Bugajev V, Halova I, Draberova L, et al. Negative regulatory roles of ORMDL3 in the FcεRI-triggered expression of proinflammatory mediators and chemotactic response in murine mast cells. *Cell Mol Life Sci.* 2016;73(6):1265-1285.

6. Bogaert P, Naessens T, De Koker S, et al. Inflammatory signatures for eosinophilic vs. neutrophilic allergic pulmonary inflammation reveal critical regulatory checkpoints. *Am J Physiol Lung Cell Mol Physiol.* 2011;300(5):L679-690.

7. Tsantikos E, Gottschalk TA, L'Estrange-Stranieri E, et al. Enhanced Lyn activity causes severe, progressive emphysema and lung cancer. *Am J Respir Cell Mol Biol.* 2023; 69(1):99-112.

8. Bonnardel E, Prevel R, Campagnac M, et al. Determination of reliable lung function parameters in intubated mice. *Respir Res.* 2019;20(1):211.
