# Supplementary material for: Tetraspanin CD53 Promotes Inflammation but Restrains Mucus Production in a Mouse Model of Allergic Airway Inflammation
Source: Allergy. 2024 Dec 9;80(4):1127–31. doi: 10.1111/all.16426 (PMC11969303; doi:10.1111/all.16426)
Supplement: Supplementary file 2 — Data S2 [file ALL-80-1127-s002.pdf]

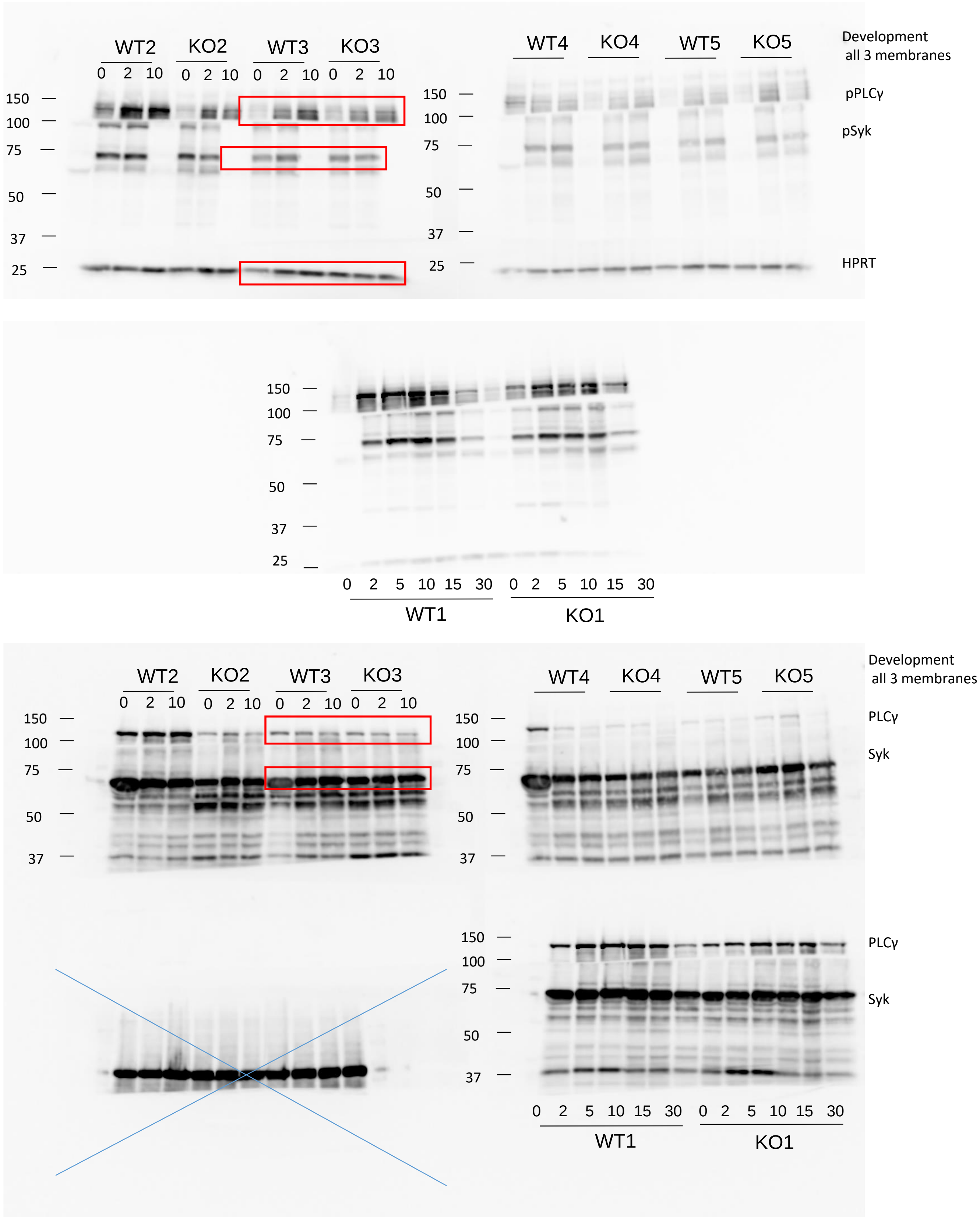

Uncropped blots 1: All immunoblot membranes for Figure S1F. Membranes were first developed for pPLCγ, pSYK and HPRT. After stripping membrane were developed for PLCγ and SYK. Five biological replicates. Red rectangles shows representative protein bands used in Figure S1F.

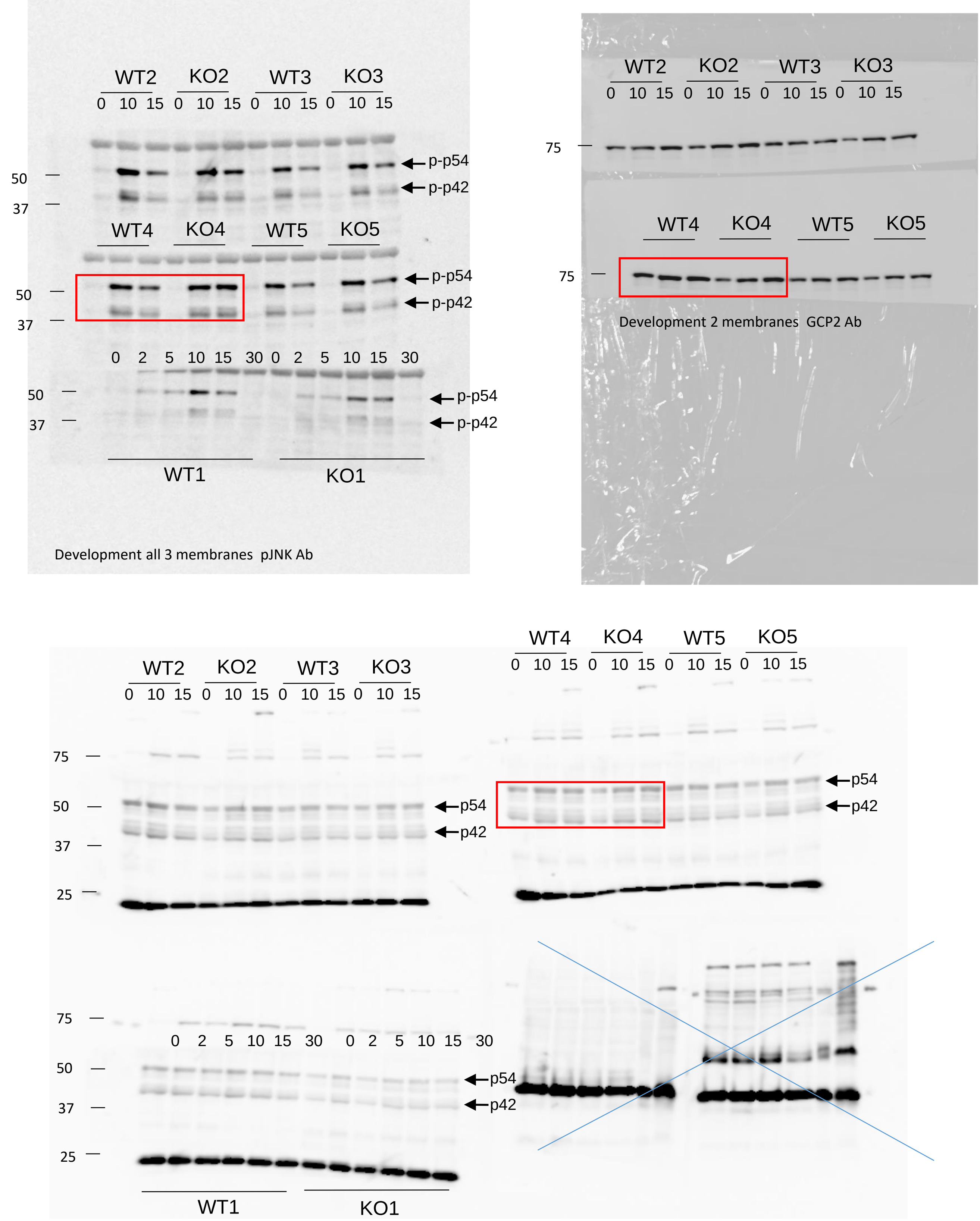

Uncropped blots 2: All immunoblot membranes for Figure 1E pJNK. Membranes were first developed for pJNK. After stripping membrane were developed for JNK and GRB2. Upper part of the membranes were developed for GCP2. Five biological replicates. Red rectangles shows representative protein bands used in Figure 2D.

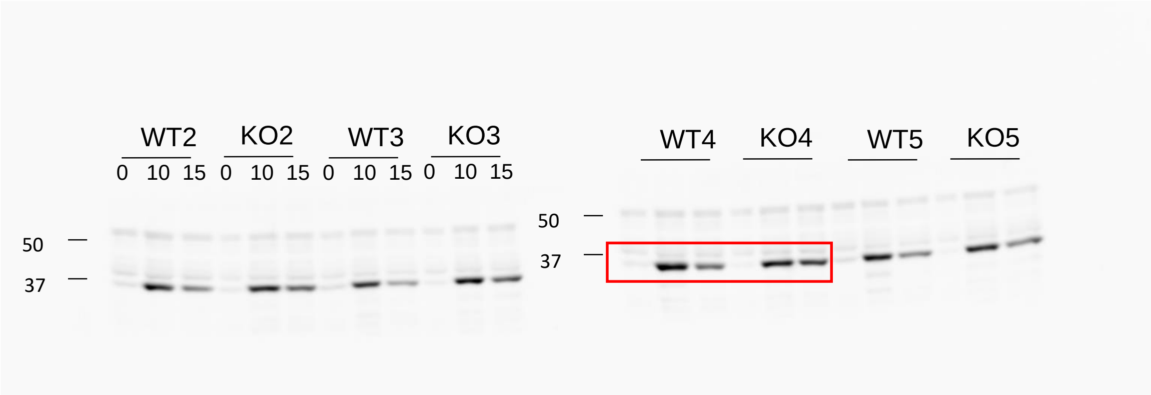

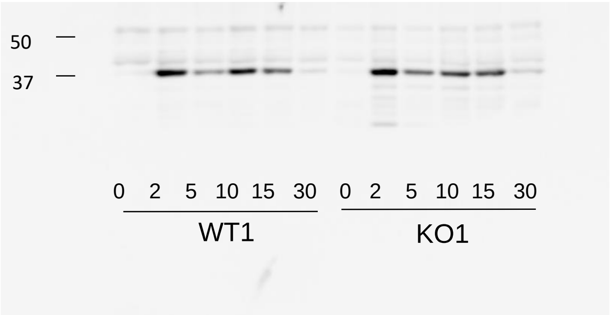

Development all 3 membranes pp38 Ab

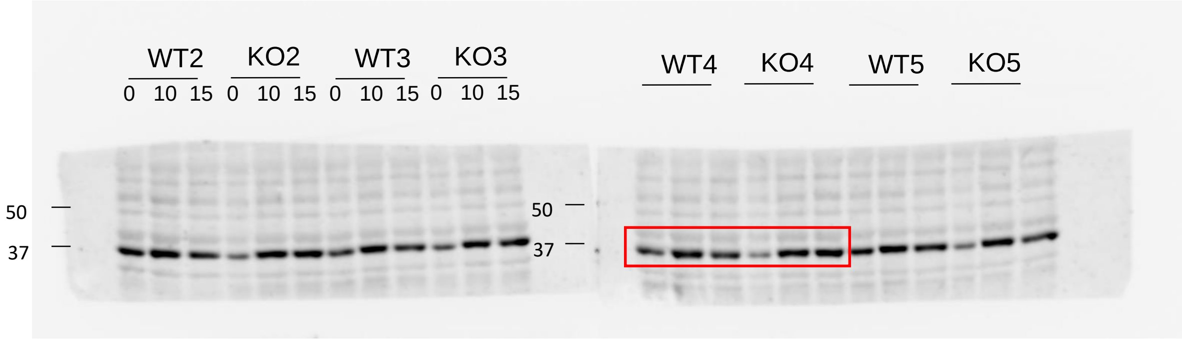

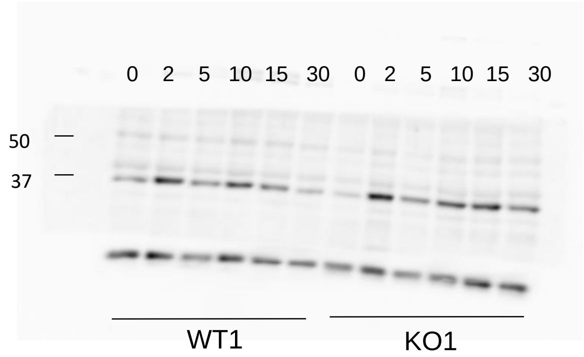

Development all 3 membranes p38 Ab

Uncropped blots 3: All immunoblot membranes for Figure 1E pp38. Middle parts of membranes shown uncropped blots 2 were stripped and first developed for pp38. After another stripping membrane were developed for p38. Five biological replicates. Red rectangles shows representative protein bands used in Figure 1E.

A

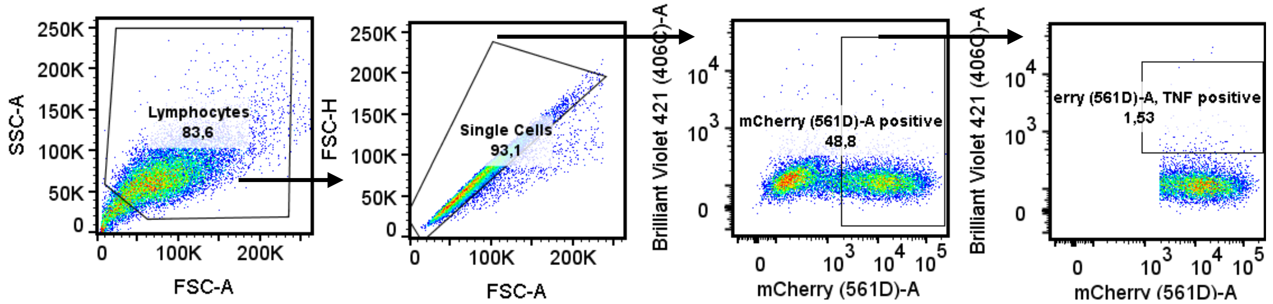

B

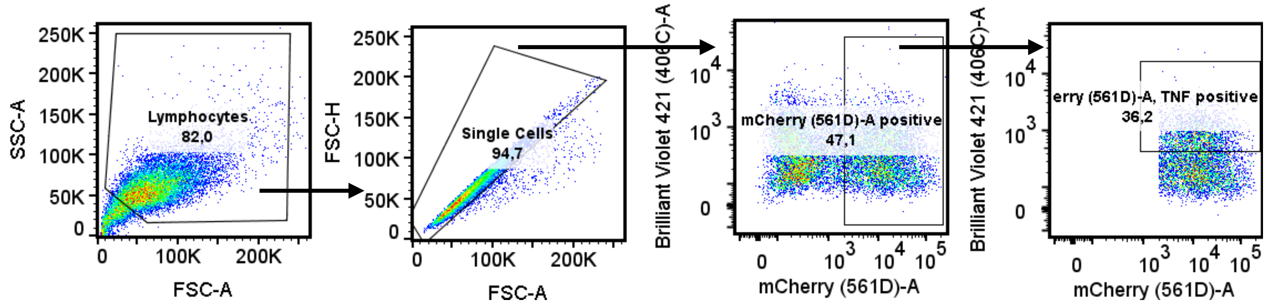

Gating strategy for CD53 „rescue" cytokine production. Single mast cells were gated for mCherry positivity. TNF-α was detected with specific BV-421 antibody. (A) non-activated mast cells, (B) activated mast cells.

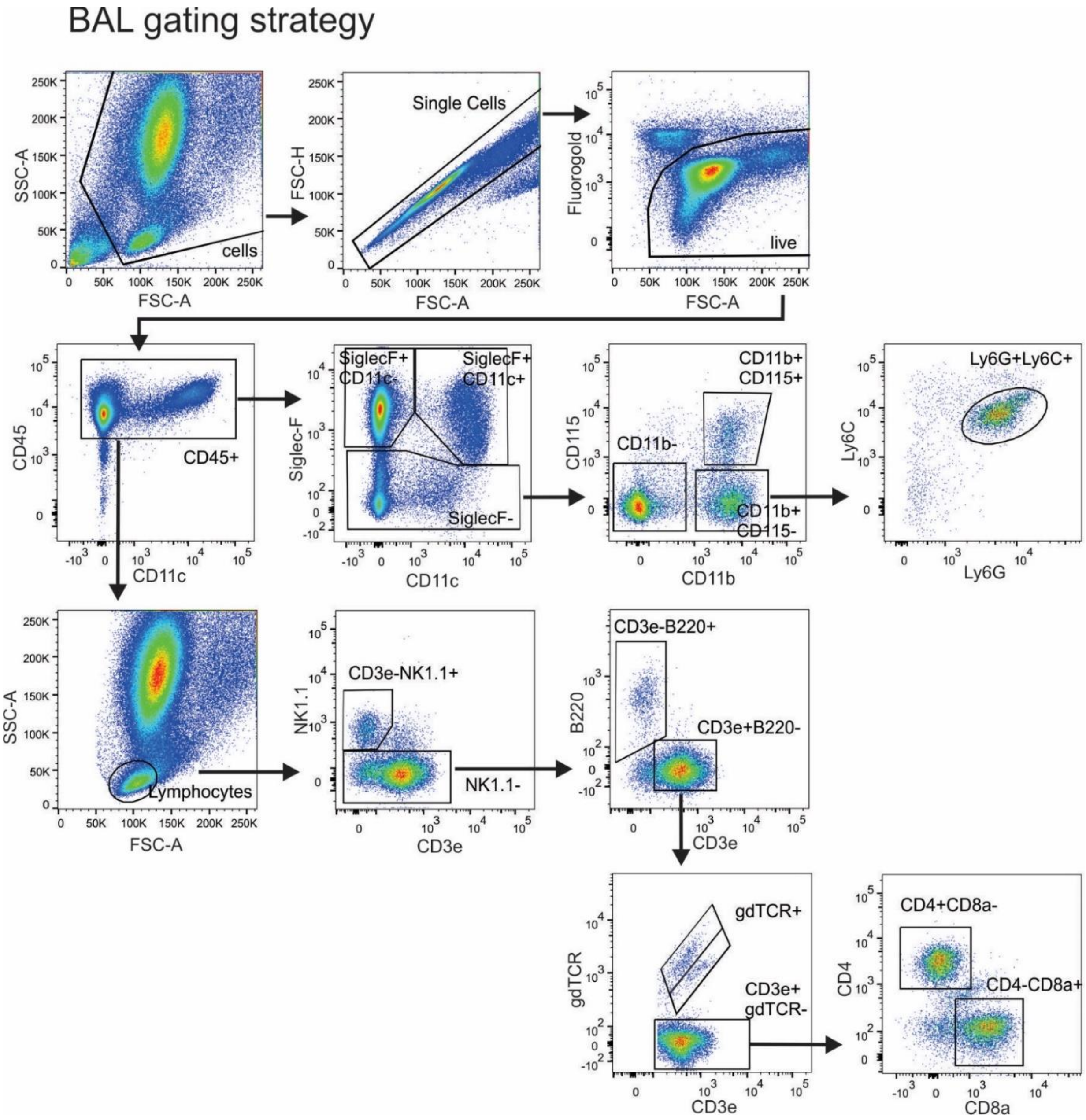

Gating strategy for BAL. Gating strategy used for flow cytometry of BAL cells. BAL cells were assessed using flow cytometry by gating on single, live, CD45+ cells. Alveolar macrophages (AMΦ) were defined as Siglec-F+CD11c+, eosinophils as Siglec-F+CD11c-, neutrophils as Siglec-F-CD11b+CD115-Ly6G+Ly6C+ and monocytes as Siglec-F-CD11b+CD115+. Lymphocytes were gated on single, live, CD45+ cells using SSC-A vs FSC-A. NK cells were distinguished in the lymphocyte gate as CD3e-NK1.1+, B cells as NK1.1-CD3e-B220+ and γδ T cells as NK1.1-CD3e+B220-γδTCR+. CD4+ T cells were defined as NK1.1-CD3e+B220-γδTCR-CD4+ and CD8+ T cells were defined as NK1.1-CD3e+B220-γδTCR-CD8a+.

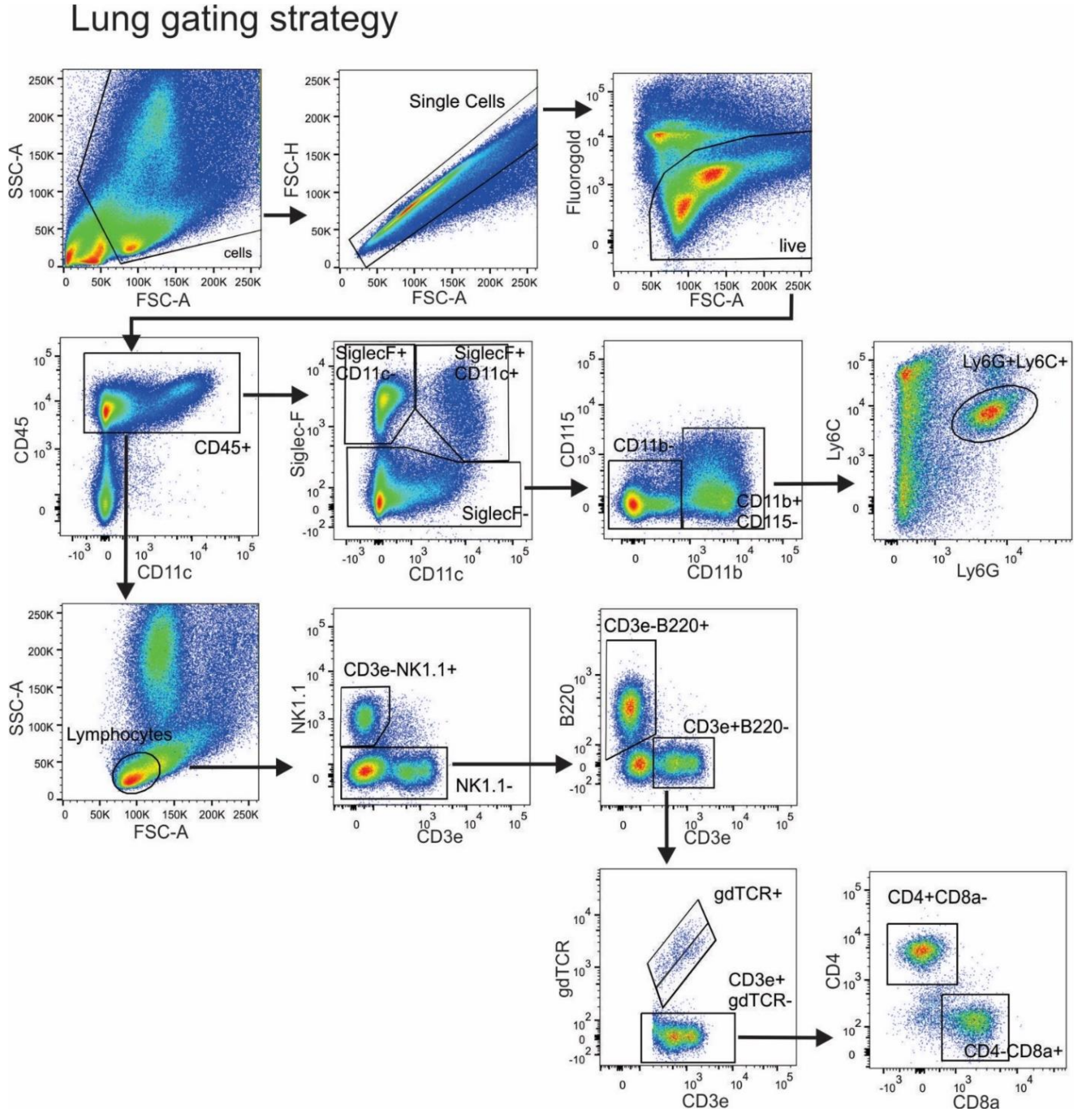

Gating strategy for lungs. Gating strategy for flow cytometry of lung tissue cells. A single cell suspension of digested lung tissue was assessed using flow cytometry by gating on single, live, CD45+ cells. Alveolar macrophages (AMΦ) were defined as Siglec-F+CD11c+, eosinophils as Siglec-F+CD11c- and neutrophils as Siglec-F-CD11b+CD115-Ly6G+Ly6C+. Lymphocytes were gated on single, live, CD45+ cells using SSC-A vs FSC-A. NK cells were distinguished in the lymphocyte gate as CD3e-NK1.1+, B cells as NK1.1-CD3e-B220+ and γδ T cells as NK1.1-CD3e+B220-γδTCR+. CD4+ T cells were defined as NK1.1-CD3e+B220-γδTCR-CD4+ and CD8+ T cells were defined as NK1.1-CD3e+B220-γδTCR-CD8a+.
